# Re-assessing the social climate of physical (in)activity in Canada

**DOI:** 10.1186/s12889-023-17436-3

**Published:** 2023-12-21

**Authors:** Matthew James Fagan, Leigh M. Vanderloo, Ananya Banerjee, Leah J. Ferguson, Eun-Young Lee, Norman O’Reilly, Ryan E. Rhodes, John C. Spence, Mark S. Tremblay, Guy Faulkner

**Affiliations:** 1 School of Kinesiology, University of British Columbia, Vancouver, BC Canada; 2 ParticipACTION, 77 Bloor Street West, Suite 1205, Toronto, ON Canada; 3 School of Occupational Therapy, Western University, London, ON Canada; 4 Department of Epidemiology, Biostatistics and Occupational Health, McGill University, Montreal, QC Canada; 5 College of Kinesiology, University of Saskatchewan, 87 Campus Drive, Saskatoon, SK Canada; 6 School of Kinesiology and Health Studies, Queen’s University, Kingston, ON Canada; 7 Graduate School of Business, University of Maine, Orono, ME USA; 8 Behavioural Medicine Laboratory, School of Exercise Science, Physical and Health Education, University of Victoria, Victoria, BC Canada; 9 Faculty of Kinesiology, Sport, and Recreation, University of Alberta, 1-153 Van Vliet Complex, Edmonton, AB Canada; 10 Children’s Hospital of Eastern Ontario Research Institute, 401 Smyth Road, Ottawa, ON Canada; 11 Department of Pediatrics, University of Ottawa, 401 Smyth Road, Ottawa, ON Canada

**Keywords:** Ecological model, Physical activity, Policy, Public opinion, Social climate

## Abstract

Social-ecological models suggest that a strategy for increasing population physical activity participation is to reconstruct the “social climate” through changing social norms and beliefs about physical activity (PA). In this study, we assessed whether the PA social climate in Canada has changed over a five-year period after controlling for sociodemographic factors and PA levels. Replicating a survey administered in 2018, a sample of adults in Canada (*n* = 2,507) completed an online survey assessing social climate dimensions, including but not limited to descriptive and injunctive norms. Descriptive statistics were calculated, and binary logistic regressions were conducted to assess the associations of sociodemographic factors and year of the survey with social climate dimensions. Results suggest some social climate constructs are trending in a positive direction between 2018 and 2023. Physical inactivity was considered a serious public health concern by 49% of respondents, second to unhealthy diets (52%). Compared to those who participated in the 2018 survey, participants in 2023 were less likely to see others walking or wheeling in their neighbourhood (OR = 1.58, 95% CI: 1.41, 1.78), but more likely to see people exercising (OR = 0.82, 95% CI: 0.73, 0.92) and kids playing in their neighbourhood (OR = 0.75, 95% CI: 0.66, 0.85). No changes were reported between 2018 and 2023 in individuals’ perceptions of whether physical inactivity is due to individual versus external factors (OR = 0.99, 95% CI: 0.87, 1.13). The findings of this work indicate a modest positive shift in some measured components of the social climate surrounding PA although attributing causes for these changes remain speculative.

## Introduction

Despite knowledge of the heightened risk of mortality and morbidity from physical inactivity, many adults living in Canada are not attaining the recommended 150 min per week of moderate-to-vigorous physical activity (MVPA) [1–3]. Recent work highlights the considerable impact of physical inactivity on the Canadian economy, suggesting that increasing physical activity (PA) and reducing sedentary behaviour would reduce healthcare costs by $2.6 billion and inject $7.5 billion into the gross domestic product of Canada by 2040 [4]. The importance of increasing population level PA cannot be overstated.

Based on social-ecological models, multi-faceted approaches have been recommended to increase PA at the population level [5–8]. Such strategies include understanding and changing the social climate surrounding health behaviours. Social climate is an umbrella term comprising the general feelings, attitudes, beliefs and opinions on a subject within a society [9]. Social norms, a pattern of behaviours or beliefs held by society [7, 10], are one of the most salient constructs of social climate [10]. General social norms can be captured through the perceived seriousness of health behaviours; however, social norms can also be further differentiated into descriptive, injunctive, and collective norms [11]. Descriptive norms attempt to describe the climate surrounding seeing the behaviour in an individual’s context (e.g., do you see others being active in your neighbourhood; [10]). Injunctive norms refer to perceptions of the acceptability of the behaviour in society (e.g., society disapproves of physical inactivity; [12]). Finally, collective norms provide insight into an individual’s perception of the political landscape, social institutions and causes of a social issue [13]. At an individual level, extensive research has demonstrated social norm associated variables to be typically weak but significant predictors of behaviour change [14–16]. Collectively, the social climate at a population level may influence individual behaviour and support for policies addressing a public health issue.

De-normalizing smoking through social marketing and changes in policy (e.g., no smoking in bars) has reduced smoking prevalence [17–20]. For example, the International Tobacco Control Europe Surveys found that awareness of anti-tobacco information was associated with feeling uncomfortable about smoking [20]. Additionally, reporting disapproval of tobacco products by people who are important to one’s life was associated with cessation attempts [20]. Research has also explored the social norms surrounding obesity. For example, Oliver and Lee [21] found that many Americans are not seriously concerned with obesity and express low support for policies addressing obesity. They further highlight that many Americans believe that obesity is caused by individual rather than societal factors. These authors concluded that as a result of these perceptions there may be challenges in enacting legislation addressing obesity [21]. Similar findings have been found in the Canadian context regarding policies for obesity with high levels of support found for individual-focused policy but low levels of support for restrictive environmental and economic policies [22].

A focus on constructs such as descriptive norms and related constructs has received much attention in the exercise psychology literature primarily as an individual correlate of physical activity [23]. Less is known about whether changing the social climate at a population level may in turn impact population levels of PA. Only one known study has attempted to quantify the social climate of physical (in)activity in Canada [24]. Yun and colleagues found that 55% of adults living in Canada agreed physical inactivity is a serious public health concern, and that 28% of participants believed that society disapproves of physical inactivity. Further, strong support was noted for environmental, individual, and economic level policy changes to promote PA. However, much less support was found in taking legislative approaches. Furthermore, demographic variables and PA levels, respectively, were associated with social norms surrounding physical (in)activity [24]. Specifically, individuals meeting the Canadian PA guidelines reported more positive social norms about PA and greater levels of support for related policies than physically inactive participants. This initial research [24] provides a benchmark that can be re-assessed over time to determine if social climate changes in response to broader policy and programmatic initiatives or whether changes in social climate precede the consideration and implementation of greater policy and legislative innovation to support population PA.

The greatest impact on social climate for PA since 2018 may have been the COVID-19 pandemic. The COVID-19 pandemic has been associated with various changes in PA across Canada [25–27]. For example, Di Sebastiano found that adults living in Canada had declines in MVPA and light physical activity (LPA) immediately following the declaration of COVID-19 [26]. However, 6-weeks later MVPA returned to pre-pandemic levels, but LPA remained lower. Data from Statistics Canada reported that between 2018 and the fall of 2020 there was a decrease in PA for youth, stability in adults (18–49 years) but significant increases for adults > 50 years [28]. Yet, the development and popularity of initiatives to support PA [28], such as closing streets or traffic lanes to cars to make more outdoor space for those who use non-vehicle users, might have heightened awareness of PA as an important health-enhancing behaviour. In response to the need for ongoing tracking of social climate constructs over time and identification of any temporal (in)stability, the purpose of this study was to explore how the social climate surrounding physical (in)activity in Canada has changed over five years after controlling for important demographic factors and PA levels.

## Methods

### Sample and recruitment

A total of 2,519 adults living in Canada were recruited from the Angus Reid Forum by MARU/Matchbox (a global market research and data science firm) in 2018 and 2,507 in 2023. As described elsewhere [24], this panel – which is comparable with the Canadian census in terms of age, sex, region, income, employment, and language spoken – includes 100,000 adults living in Canada who have already consented to participate in research. From this panel of 100,000 adults, MARU contacts eligible participants until the sample size is met with no missing data. The response rate for this study was approximately 25%. Data collection in 2018 and 2023 was conducted by the not-for-profit PA promotion organization ParticipACTION (www.participaction.com). The secondary data analysis was approved by the University of British Columbia Institutional Behavioural Research Ethics Board (#H23-00510).

### Data collection procedures

Repeated cross-sectional data were collected in 2018 and 2023 through surveys deployed online in French and English. In 2018, the survey was opened on January 15 and remained open for seven days. In 2023, the survey opened on February 27 and remained open for seven days. In both instances of data collection, participants were sent an email with a one-time link to the survey. A reminder email was sent two days before the survey closed. The survey required approximately 15 min to complete. Once closed, all data were cleaned, de-identified, and tabulated into an SPSS file (version 27, IBM, New York, USA).

### Measures

#### Survey development procedure

The initial survey was created with guidance from the obesity and tobacco control literature on social climate (most notably the Tobacco Control Survey). Once developed in 2018, the survey was reviewed by two advisory groups within ParticipACTION for feedback and face validity assessment. In 2018, the initial survey had high test-retest reliability for all measures (ICC = 0.70) except one item (society disapproves of PA ICC = 0.33) [24]. The revised survey deployed in 2023 had some minor changes which are highlighted in the following sections.

##### Seriousness of public health issues.

The perceived seriousness of different health risk behaviours was assessed based on previous work with obesity [21]. In 2018, the health behaviours included physical (in)activity, sitting too much among those who are able-bodied (sedentary behaviour), tobacco use, alcohol misuse, cannabis use, unhealthy diets, and lack of sleep. In 2023, two health behaviours were added; e-cigarette use and excessive recreational screen time. Respondents were asked to rate perceived seriousness on a 7-point scale ranging from “not at all serious [1]” to “very serious [7].” For analysis purposes, the seriousness of physical (in)activity was coded into two groups; (1) *not serious* (1–5 on a 7-point scale) and (2) *serious* (6–7 on a 7-point scale). These groups are consistent with previous work on the social climate of physical activity [24].

##### Descriptive norms.

Consistent with the baseline study [24], five descriptive norms were assessed in 2023. These items were modified from previous work in the obesity literature [24, 29]. The items were: “*I often see other people walking or wheeling in my neighbourhood*”, “*I often see other people exercising (e.g., jogging, bicycling, playing sports) in my neighbourhood*”, and “*I often see kids playing actively (e.g., playing games like tag, sports, riding their bikes) in my neighbourhood*”. Response options for each item were on a 7-point scale ranging from “strongly disagree [1]” to “strongly agree [7]”. To be consistent with the multinomial logistic regression approach taken in 2018 [10], two categories were created to reflect (1) agree (*strongly agree, moderately agree*) and (2) disagree (*slightly agree, neutral, slightly disagree, disagree, strongly disagree)* [24]. Additionally, respondents were asked to estimate the proportion of adults their age meeting PA guidelines on a scale ranging from 0 to 100% in increments of 10%. For analytical purposes, this variable was collapsed into two groups: 1) ≤ 50% and 2) > 50%.

##### Injunctive norms.

Injunctive norms were assessed through two items. The first assessed the acceptability of physical inactivity: “*Society disapproves of physical inactivity*.” Responses on the item were made on a 7-point scale ranging from “strongly disagree [1]” to “strongly agree [7]”. Two categories were created to reflect (1) agree (*strongly agree, moderately agree*) and (2) disagree (*slightly agree, neutral, slightly disagree*, disagree, *strongly disagree*). These groupings were based on previous work on the social climate of physical activity [24].

The second injunctive norm assessed how many people close to you are meeting Canadian guidelines for PA “*How many people who are important to you (e.g., friends or family) would you say engage in 150 minutes of moderate-to-vigorous physical activity per week?*” This item was rated on a 5-point scale ranging from [1] “*all of them*” to “*none of them*” [5] with “*some of them*” in the middle [3]. Two categories were created to reflect (1) all of them/most of them and (2) some to none of them.

##### Collective norms: Perceptions of the causes of physical (in)activity.

Collective norms were assessed through the perceptions of the causes of physical (in)activity consistent with the baseline study [24]. Participants were asked to select one of the following options regarding physical inactivity being “*an individual’s fault*”, “*caused by other factors beyond an individual’s control*”, “*both an individual’s fault and caused by other factors beyond an individual’s control*”, “*neither an individual’s fault nor caused by other factors beyond an individual’s control*”, and “*don’t know*”. For analysis, two categories were used for the causes of physical (in)activity; (1) solely individual (only including “*an individual’s fault*”) or (2) external factors (includes all other responses).

##### Physical activity.

PA was assessed with the PA for Adults Questionnaire (PAAQ) in 2018 [24, 30]. The PAAQ has been shown to have adequate validity and reliability [30]. Additionally, a dichotomous measure was created to reflect meeting 150 min of moderate to vigorous PA (MVPA) per week [24]. In 2023, a single item captured PA participation [31, 32]. This measure asks, “*In the past week, on how many days have you done a total of 30 minutes or more of physical activity, which was enough to raise your breathing rate? This may include sport, traditional games, exercise, and brisk walking or cycling for recreation or to get to and from places, but should not include housework or physical activity that may be part of your job*.”. The item is then scored on a 0–7-day scale. This measure has been shown to have adequate reliability and validity for overall PA levels [31]. Once collected, individuals reporting five or more days were coded as meeting the PA guidelines in line with national guidelines [1, 31].

##### Demographics.

Several demographic variables were measured and used for covariates in our analysis. Respondents reported gender (*woman, man*), age, and dwelling (*urban, semi-urban, rural*), household income (*<$35,000, $35,000-$75,000, $75,000-$125,000, >$125,000*) and political orientation (*Liberal, Centre, Conservative* and *I don’t know*).

### Statistical analysis

Descriptive and inferential statistics were used to describe and assess the differences in demographic variables and health behaviours for dimensions of the social climate of physical (in)activity in 2023 (Student’s t-test for continuous variables and the Chi-squared test for categorical variables) [33]. All analyses were conducted using R (version 4.2.1).

Binary logistic regression models were built using the *stats* library and the *glm* function in *R*. These eight separate models were created to reflect general social norms (i.e., “*the seriousness of physical (in)activity*”), descriptive norms (i.e., “*I see people walking or wheeling in my neighbourhood”, “I see people exercising in my neighbourhood”, “I see children playing in my neighbourhood”*, and *“How many people your age* *meet the Canadian Physical Activity Guidelines”*), injunctive norms (i.e., “*Society disapproves of physical (in)activity*” and *“How many people important to you* *about meet the Canadian Physical Activity Guidelines*”) and collective norms (i.e., *“Responsibility of physical (in)activity”*). In all models, gender, age, dwelling setting, political orientation, and MVPA status were covariates based on previous work [24, 34]. After constructing models, observed power was calculated and is presented within model tables through the *powerSim* function in the *pwr* library. The calculations are based on 1000 simulations, alpha = 0.05 and the sample size of 5,016. The results presented in Tables 1, 2, 3 and 4 include the observed power for the predictor Time.

**Table 1:** Seriousness of physical (in)activity

Characteristic	OR^1^	95% CI^1^	*p*-value
**Gender**			
Men	Ref	—	
Women	1.31	1.17, 1.48	**< 0.001**
**Age**	1.01	1.00, 1.01	**0.002**
**Dwelling setting**			
I live in an urban setting	Ref	—	
I live in a semi-urban setting	0.93	0.82, 1.06	0.3
I live in a rural setting	0.98	0.84, 1.15	0.8
**Income level ($)**			
< 35,000	Ref	—	
35,000–75,000	1.15	0.97, 1.36	0.10
75,000-125,000	1.32	1.11, 1.57	**0.002**
> 125,000	1.30	1.07, 1.59	**0.009**
Don’t want to report	1.30	1.06, 1.60	**0.011**
**Met the physical activity guidelines**			
No	Ref	—	
Yes	1.59	1.38, 1.83	**< 0.001**
**Political Spectrum**			
Liberal	Ref	—	
Centre	0.88	0.76, 1.01	0.071
Conservatives	0.85	0.73, 0.99	**0.036**
Don’t know	0.85	0.71, 1.02	0.085
**Year**			
2018	Ref	—	
2023	0.75	0.66, 0.84	**< 0.001**

^1^ OR = Odds Ratio, CI = Confidence Interval, Ref = Reference group for each corresponding variable

Outcome reference group: Not serious

Observed power (1- β) = 99.8, 95% CI: 99.6, 100.0

**Table 2:** Descriptive norms

Walking or Wheeling^2^				People exercising^2^			Kids playing^2^			People my age.guidelines^2^		
Characteristic	OR^1^	95% CI^1^	*p*-value	OR^1^	95% CI^1^	*p*-value	OR^1^	95% CI^1^	*p*-value	OR^1^	95% CI^1^	*p*-value
**Gender**												
Male	REF	—		—	—		—	—		—	—	
Female	0.77	0.69, 0.87	**< 0.001**	0.79	0.70, 0.88	**< 0.001**	0.86	0.75, 0.97	**0.017**	1.14	0.98, 1.32	0.10
**Age**	0.99	0.99, 0.99	**< 0.001**	1.00	0.99, 1.00	0.4	1.01	1.01, 1.01	**< 0.001**	0.98	0.97, 0.98	**< 0.001**
**Dwelling setting**												
I live in an urban setting	REF	—		—	—		—	—		—	—	
I live in a semi-urban setting	1.05	0.92, 1.20	0.4	1.05	0.92, 1.20	0.5	0.97	0.85, 1.12	0.7	0.97	0.83, 1.15	0.7
I live in a rural setting	1.71	1.46, 2.00	**< 0.001**	1.58	1.35, 1.86	**< 0.001**	1.36	1.14, 1.63	**< 0.001**	0.80	0.65, 0.98	**0.037**
**Income level ($)**												
< 35,000	REF	—		—	—		—	—		—	—	
35,000–75,000	1.05	0.89, 1.25	0.6	0.88	0.74, 1.04	0.13	1.03	0.85, 1.23	0.8	1.04	0.85, 1.29	0.7
75,000-125,000	1.02	0.86, 1.22	0.8	0.95	0.79, 1.13	0.5	1.01	0.83, 1.22	> 0.9	0.79	0.63, 0.99	**0.039**
> 125,000	0.99	0.81, 1.21	> 0.9	0.86	0.70, 1.05	0.13	0.98	0.79, 1.22	0.9	0.77	0.60, 0.99	**0.041**
Don’t want to report	0.99	0.80, 1.21	0.9	1.05	0.86, 1.30	0.6	1.20	0.95, 1.51	0.12	0.89	0.68, 1.16	0.4
**Met the physical activity guidelines**												
No	REF	—		—	—		—	—		—	—	
Yes	0.83	0.72, 0.95	**0.007**	0.71	0.62, 0.82	**< 0.001**	0.72	0.63, 0.84	**< 0.001**	1.58	1.34, 1.86	**< 0.001**
**Political Spectrum**												
Liberal	REF	—		—	—		—	—		—	—	
Centre	1.36	1.18, 1.57	**< 0.001**	1.08	0.93, 1.25	0.3	0.99	0.85, 1.15	0.9	1.12	0.94, 1.35	0.2
Conservatives	1.17	1.01, 1.36	**0.041**	1.09	0.93, 1.26	0.3	1.09	0.92, 1.28	0.3	1.05	0.86, 1.29	0.6
Don’t know	1.35	1.13, 1.62	**0.001**	1.19	0.99, 1.44	0.058	1.29	1.05, 1.58	**0.014**	1.02	0.81, 1.28	0.8
**Year**												
2018	REF	—		—	—		—	—		—	—	
2023	1.58	1.41, 1.78	**< 0.001**	0.82	0.73, 0.92	**< 0.001**	0.75	0.66, 0.85	**< 0.001**	2.20	1.89, 2.56	**< 0.001**

^1^ OR = Odds Ratio, CI = Confidence Interval, REF = Reference group for each corresponding variable

^2^Outcome reference group: Disagree, ^3^ Outcome reference group < = 50%

Observed power (1- β) (walking or wheeling) = 99.8, 95% CI: 99.6, 100.0, Observed power (1- β) (people exercising) = 90.0, 95% CI: 87.9, 91.8, Observed power (1- β) (kids playing) = 99.5, 95% CI: 98.8, 99.8, Observed power (1- β) (people my age) = 99.8, 95% CI: 99.6, 100.0

**Table 3:** Injunctive norms

Society disapproves of inactivity^2^				People I care about…guidelines ^3^		
Characteristic	OR^1^	95% CI^1^	*p*-value	OR^1^	95% CI^1^	*p*-value
**Gender**						
Male	REF	—		—	—	
Female	0.72	0.63, 0.82	**< 0.001**	1.19	1.02, 1.38	**0.030**
**Age**	1.00	1.00, 1.00	0.6	1.02	1.01, 1.02	**< 0.001**
**Dwelling setting**						
I live in an urban setting	REF	—		—	—	
I live in a semi-urban setting	1.16	1.00, 1.35	**0.044**	1.33	1.12, 1.58	**0.001**
I live in a rural setting	1.11	0.93, 1.32	0.3	1.14	0.93, 1.42	0.2
**Income level ($)**						
< 35,000	REF	—		—	—	
35,000–75,000	1.09	0.90, 1.31	0.4	1.09	0.87, 1.35	0.4
75,000-125,000	1.09	0.90, 1.33	0.4	1.25	0.99, 1.57	0.060
> 125,000	1.10	0.88, 1.37	0.4	1.16	0.90, 1.50	0.2
Don’t want to report	1.08	0.86, 1.36	0.5	0.97	0.74, 1.28	0.8
**Met the physical activity guidelines**						
No	REF	—		—	—	
Yes	1.03	0.88, 1.20	0.8	0.43	0.37, 0.51	**< 0.001**
**Political Spectrum**						
Liberal	REF	—		—	—	
Centre	1.29	1.11, 1.52	**0.001**	1.12	0.93, 1.36	0.2
Conservatives	1.59	1.34, 1.90	**< 0.001**	1.12	0.92, 1.38	0.3
Don’t know	1.75	1.42, 2.16	**< 0.001**	1.31	1.02, 1.67	**0.033**
**Year**						
2018	REF	—		—	—	
2023	1.17	1.03, 1.34	**0.015**	0.58	0.50, 0.68	**< 0.001**

^1^ OR = Odds Ratio, CI = Confidence Interval, REF = Reference group for each corresponding variable

^2^Outcome reference group: Disagree, ^3^ Outcome reference group: all or most of them

Observed power (1- β) (society disapproves) = 69.5, 95% CI: 66.5, 72.3,

Observed power (1- β) (People I care about) = 99.8, 95% CI: 99.6, 100.0

**Table 4:** Collective norms: perceptions of the causes of physical (in)activity

Characteristic	OR^1^	95% CI^1^	*p*-value
**Gender**			
Male	—	—	
Female	1.33	1.17, 1.52	**< 0.001**
**Age**	0.99	0.99, 0.99	**< 0.001**
**Dwelling setting**			
I live in an urban setting	—	—	
I live in a semi-urban setting	1.02	0.88, 1.18	0.8
I live in a rural setting	0.80	0.68, 0.95	**0.011**
**Income level ($)**			
< 35,000	—	—	
35,000–75,000	0.75	0.61, 0.91	**0.003**
75,000-125,000	0.60	0.49, 0.74	**< 0.001**
> 125,000	0.62	0.49, 0.78	**< 0.001**
Don’t want to report	1.02	0.80, 1.31	0.9
**Met the physical activity guidelines**			
No	—	—	
Yes	0.68	0.58, 0.79	**< 0.001**
**Political Spectrum**			
Liberal	—	—	
Centre	0.59	0.50, 0.70	**< 0.001**
Conservatives	0.50	0.42, 0.59	**< 0.001**
Don’t know	0.59	0.48, 0.73	**< 0.001**
**Year**			
2018	—	—	
2023	0.99	0.87, 1.13	0.9

^1^ OR = Odds Ratio, CI = Confidence Interval, REF = Reference group for each corresponding variable

Outcome reference group: Individual is responsible

Observed power (1- β) = 5.00, 95% CI: 3.73, 6.54

## Results

### Missing data and data exclusions

There were no missing data for 2018 or 2023. Individuals who identified as nonbinary, two-spirit, preferred not to answer or did not know their gender were excluded from our analysis as they represented less than 1% of the sample.

### Descriptive results

The sociodemographic results between 2018 and 2023 are presented in Table 5. In terms of demographic differences between 2018 and 2023, the sample in 2023 had a higher percentage of women, was on average younger, and included more individuals living in a suburban setting. In 2023, 49% of individuals felt that physical (in)activity was a serious concern. This was similar to unhealthy diets (52%), alcohol misuse (49%), tobacco use (47%), and e-cigarette use (46%). 45% of participants agreed with seeing people walking or wheeling in their neighbourhood, 46% agreed with seeing others exercising, and 34% with seeing kids playing in their neighbourhood. 22% indicated all or most of the people important to them were meeting the Canadian guidelines for PA, and 26% agreed that society disapproves of physical inactivity. Finally, 28% of participants reported that they believe it is an individual’s fault for physical inactivity in Canada.

**Table 5:** Participant characteristics by year

Characteristics	2018, N = 2,519^1^	2023, N = 2,497^1^	*p*-value
**Demographics**			
**Gender** ^**1**^			< 0.001^4^
Men	1,266 (50%)	1,021 (41%)	
Women	1,253 (50%)	1,476 (59%)	
**Age** ^**2**^	49 (16)	47 (17)	0.001^3^
**Dwelling setting** ^**1**^			< 0.001^4^
I live in an urban setting	1,363 (54%)	1,237 (50%)	
I live in a semi-urban setting	668 (27%)	850 (34%)	
I live in a rural setting	488 (19%)	410 (16%)	
**Income ($)** ^**1**^			< 0.001^4^
< 35,000	407 (16%)	520 (21%)	
35,000–75,000	719 (29%)	749 (30%)	
75,000-125,000	630 (25%)	590 (24%)	
> 125,000	331 (13%)	405 (16%)	
Don’t want to report	432 (17%)	233 (9.3%)	
**Met the physical activity guidelines** ^**1**^			< 0.001^4^
No	2,110 (84%)	1,815 (73%)	
Yes	409 (16%)	682 (27%)	
**Political Spectrum** ^**1**^			< 0.001^4^
Liberal	885 (35%)	919 (37%)	
Centre	565 (22%)	766 (31%)	
Conservatives	693 (28%)	491 (20%)	
Don’t know	376 (15%)	321 (13%)	
**Issue Importance**			
**Tobacco use importance** ^**1**^			< 0.001^4^
Not serious	1,087 (43%)	1,335 (53%)	
Serious	1,432 (57%)	1,162 (47%)	
**Alcohol misuse importance** ^**1**^			0.3^4^
Not serious	1,253 (50%)	1,279 (51%)	
Serious	1,266 (50%)	1,218 (49%)	
**Not Enough Physical activity importance** ^**1**^			< 0.001^4^
Not serious	1,132 (45%)	1,268 (51%)	
Serious	1,387 (55%)	1,229 (49%)	
**Cannabis use importance** ^**1**^			0.7^4^
Not serious	1,679 (67%)	1,675 (67%)	
Serious	840 (33%)	822 (33%)	
**Sitting too much importance** ^**1**^			0.9^4^
Not serious	1,543 (61%)	1,535 (61%)	
Serious	976 (39%)	962 (39%)	
**Unhealthy diets importance** ^**1**^			< 0.001^4^
Not serious	1,065 (42%)	1,211 (48%)	
Serious	1,454 (58%)	1,286 (52%)	
**Lack of sleep importance** ^**1**^			0.4^4^
Not serious	1,475 (59%)	1,430 (57%)	
Serious	1,044 (41%)	1,067 (43%)	
**E-cigarette use importance** ^**1**^			
Not serious	0 (NA%)	1,349 (54%)	
Serious	0 (NA%)	1,148 (46%)	
Unknown	2,519	0	
**Excessive recreational screen time importance** ^**1**^			
Not serious	0 (NA%)	1,392 (56%)	
Serious	0 (NA%)	1,105 (44%)	
Unknown	2,519	0	
**Descriptive norms**			
**I often see people walking** ^**1**^			< 0.001^4^
Agree	1,406 (56%)	1,136 (45%)	
Disagree	1,113 (44%)	1,361 (55%)	
**I often see others exercising** ^**1**^			< 0.001^4^
Agree	989 (39%)	1,148 (46%)	
Disagree	1,530 (61%)	1,349 (54%)	
**I often see kids playing** ^**1**^			< 0.001^4^
Agree	647 (26%)	842 (34%)	
Disagree	1,872 (74%)	1,655 (66%)	
**What percent your age meet the guidelines** ^**1**^			< 0.001^4^
0%	27 (1.1%)	91 (3.6%)	
10%	274 (11%)	221 (8.9%)	
20%	441 (18%)	294 (12%)	
30%	654 (26%)	399 (16%)	
40%	436 (17%)	436 (17%)	
50%	363 (14%)	401 (16%)	
60%	184 (7.3%)	293 (12%)	
70%	93 (3.7%)	173 (6.9%)	
80%	32 (1.3%)	93 (3.7%)	
90%	8 (0.3%)	26 (1.0%)	
100%	7 (0.3%)	70 (2.8%)	
**Injunctive norms**			
**How many people important to you meet Canadian Guidelines** ^**1**^			< 0.001^4^
All of them	62 (2.5%)	164 (6.6%)	
Most of them	272 (11%)	389 (16%)	
Some of them	919 (36%)	831 (33%)	
A few of them	1,014 (40%)	811 (32%)	
None of them	252 (10%)	302 (12%)	
**Society disapproves of physical inactivity** ^**1**^			0.091^4^
Agree	706 (28%)	647 (26%)	
Disagree	1,813 (72%)	1,850 (74%)	
**Collective Norms**			
**Perception of causes of physical inactivity** ^**1**^			< 0.001^4^
Individual’s fault	711 (28%)	703 (28%)	
External factors	1,808 (72%)	1794 (72%)	

^1^ n (% of yearly sample); ^2^Mean (SD)

^3^ Student’s t-test; ^4^Mann-Whitney Test

### Regression model results

For the impact of time (year of survey) on all models, data from 2018 were set as the referent group for each predictor variable.

#### Seriousness of public health issues: physical (in)activity

After adjusting for demographic factors and PA levels, individuals in 2023 had 25% lower odds of reporting serious concern surrounding physical (in)activity compared to 2018 (see Table 1 for full model results).

#### Descriptive norms: perceptions of PA

Overall, compared to 2018, individuals in 2023 were at lower odds of seeing others walking or wheeling in their neighbourhood but higher odds of seeing people exercising and kids playing. After adjusting for demographic factors and PA levels, individuals in 2023 had 58% higher odds of disagreeing than agreeing when considering seeing others walking or wheeling in their neighbourhood. Individuals in 2023 were at approximately 18% lower odds of disagreeing compared to agreeing with seeing people exercising in their neighbourhood. Participants in 2023 were at approximately 25% lower odds of disagreeing than agreeing with seeing kids playing in their neighbourhood. Individuals in 2023 had a 2.2-fold increase in odds of reporting that > 50% of people their age met the Canadian guidelines compared to ≤ 50% (see Table 2 for all descriptive norm models).

#### Injunctive norms: Society disapproves of physical (in)activity/people you care about meeting guidelines

After controlling for demographic factors and PA levels, individuals in 2023 were at 17% higher odds of reporting disagreeing that society disapproves of physical (in)activity than agreeing. Individuals in 2023 had 42% lower odds of reporting none or a few when considering the relative amount of people close to them meeting the Canadian PA guidelines (see Table 3 for injunctive norm models).

#### Collective norms: perceptions of causes of physical (in)activity

After controlling for demographic factors and PA levels, for individuals in 2023, there were no differences in results of reporting individual versus external factors as the cause(s) of physical (in)activity (see Table 4).

#### Impact of covariates on the models

Several covariates included in the models were associated with the social climate variables. Individuals who identify as women, live in an urban setting, met the PA guidelines or were Liberal leaning were typically at greater odds of reporting positive social norms surrounding physical (in)activity (see Tables 1, 2, 3 and 4).

## Discussion

Our work provides the first re-examination of Canada’s social climate surrounding physical (in)activity building on previously published research in 2018 [24]. Measured components of the social climate have shifted in both positive and negative directions from 2018 to 2023. Among our survey participants, 49% of participants reported physical inactivity was a serious health concern in 2023, which was higher than that of tobacco use (47%) and similar to alcohol misuse (49%). Less than half of the participants reported seeing people walking or wheeling, exercising, and kids playing in their neighbourhoods (45%, 46% and 34%, respectively). 26% of the sample agreed that society disapproves of physical inactivity, and only a minority (28%) of the participants saw the cause of physical inactivity to be solely down to the individual.

When considering seriousness of physical (in)activity or descriptive norms, the majority (3/5) of models indicated a more favourable reporting of social norms surrounding PA in 2023 compared to 2018. This modest shift could indicate that public awareness campaigns, policies, and/or strategies for improving PA are impacting the thoughts of people living in Canada. Alternatively, awareness of healthy living behaviours made prominent through the pandemic may have shifted social norms. Regardless, there were some conflicting findings. For example, compared to 2018, individuals were at lower odds of indicating physical (in)activity as a serious health concern. Despite this finding within our model (Table 1), our descriptive results still demonstrate that many Canadian (49% compared to 55% in 2018) report physical (in)activity as a serious public health concern. Although not assessed, it may be that other health concerns regarding COVID-19 or opioid use for example, were more salient for survey participants in 2023. Alternatively, as shown in Table 5, more people self-reported meeting national PA recommendations [1]. Data in the first year of pandemic did suggest increases among Canadians > 50 years of age [27]. If people perceive more individuals living in Canada as physically active, then perhaps intuitively, one might perceive physical (in)activity as less of a serious public health issue at a population level.

Another unexpected finding was a downward shift in the proportion of participants seeing people walking or wheeling in their neighbourhood (56% in 2018 to 45% in 2023). In response to the COVID-19 pandemic, several public health measures were introduced, including the closing of worksites, schools, and recreation programming. There were also initiatives to close roads and enhance access for walking and cycling [35–38] to help facilitate PA. However, based on meta-analyses, physical activity during COVID-19 generally declined among youth and adults [39, 40]. In contrast, a two-step literature review, including a scoping review and a narrative review, reported an increase in recreational bike trips and walking activities in the first year of the COVID-19 pandemic [38]. The discrepancy between the results from our work and previous literature may be that, in 2023, three years after the pandemic and the return to work and school, walking (or wheeling) in the neighbourhood was not as salient. Another possible explanation may be that the frame of reference has shifted, as many individuals were accustomed to seeing people walking or wheeling during the start of the pandemic. With the return to “normal” from the pandemic it is possible a small downturn in walking or wheeling from the new reference influenced perceptions. Finally, it should be noted that in 2023, a slight modification was added to the relevant survey question to make it more inclusive. In 2018 the question asked, “I often see other people walking in my neighbourhood” while in 2023 it was revised to “I often see other people walking or wheeling in my neighbourhood”.

In contrast, participants reported seeing more people exercising and more children playing in their neighbourhood. These results may reflect a long-term consequence of COVID-19 measures where exercise facilities were closed and many people continued to find other ways to exercise (e.g., running or outdoor workouts), and a renewed sense of health consciousness in light of public health messaging about the benefits of PA [41]. A change in terms of seeing children playing in the neighbourhood may be a similar consequence in some way of the pandemic but perhaps reinforced by concerted advocacy and funding efforts in Canada to support communities in increasing children’s opportunities for outdoor play [42].

The overall findings for injunctive norms were mixed. First, it was found that individuals in 2023 were at higher odds of indicating all or most of the people they care about are meeting the Canadian PA guidelines compared to 2018. An assumption here is that this might be associated with greater social pressure or expectation to engage in PA (at least in circumstances where one values the thoughts of people you care about) [12]. However, there was incongruence when considering perceptions regarding whether society disapproves of physical (in)activity, where individuals were more likely to disagree with that statement in 2023 compared to 2018. Change over the last five years was modest, with approximately 26% of participants agreeing that society disapproves of physical inactivity (compared to 28% in 2018). The implications of these social climate outcomes as they relate to physical inactivity at a population level remain speculative. The approach to tobacco use is commonly suggested as a successful normative approach in de-normalizing smoking and lowering its social acceptability [20]. Whether physical inactivity can be deemed as socially unacceptable at a population level in a similar way that encourages individual behaviour change remains to be seen.

In terms of collective norms, there was no change over five years in the proportion of adults living in Canada reporting that physical (in)activity was caused by individual factors. Reflecting social ecological models of behaviour, this remains a positive finding in that most Canadians are likely receptive to policy and practice interventions that go beyond an individual focus on education or motivation [9]. Within this model (Table 4), identifying as a man, conservative, and reporting higher income were at higher odds of reporting individuals were responsible for the causes of physical (in)activity. This finding is consistent with earlier research examining the causal attribution of physical (in)activity and political orientation, where conservatives were more likely to report internal causes for (in)activity [34]. It has been identified that political orientation moderates the framing effects of policy delivery [43]. For example, it was found that individuals who identified as conservative favoured a government benefit (for energy saving home improvements) when conceptualized as a “tax rebate” over a “subsidy” [43]. Advocacy and messaging for progressive policy implementation in the PA arena will need to be considerate of the broad political spectrum of the Canadian population [30].

### Strengths and limitations

There are several strengths to this study. First, this is the first known work to re-assess the social climate of physical (in)activity internationally. Second, the sample size in 2018 and 2023 are large and broadly representative of the Canadian population in terms of age, gender, and geographical distribution. Third, the survey deployment was provided at the same time of year to mitigate the seasonal variation in physical activity [44]. However, this work is not without limitations. The survey deployed in 2023 contained slightly modified questions from the 2018 version which may have influenced the presented findings. The cross-sectional design of this work limits the ability to infer causation, and relying on self-report increases the probability of several types of bias. Attributing causes for the changes between 2018 and 2023 remain speculative. It may be due to the success of public health campaigns and funding from the government to promote PA [3, 45]. The COVID-19 pandemic also contributed to an environment where new behaviours and social norms were created, partly out of necessity [46, 47]. Alternatively, sampling differences in the composition of the two samples may have contributed in some way to the identified changes over time.

## Conclusion

Overall, the findings of this work indicate a modest positive shift in some measured components of the social climate surrounding physical (in)activity between 2018 and 2023 in Canada. Physical inactivity remains a serious public health issue, more people in Canada report seeing others exercising and children playing in their neighbourhoods, and a higher proportion of participants report people important to them are meeting national PA guidelines. Continued monitoring is required to understand the stability of these measured constructs with consideration given as to how to assess the likely bidirectional relationship between PA and the social climate at a population level.

## Data Availability

The dataset used and analysed during the current study is available from the corresponding author on reasonable request.
